# Remarkable Sensitivity of Young Honey Bee Workers to Multiple Non-photic, Non-thermal, Forager Cues That Synchronize Their Daily Activity Rhythms

**DOI:** 10.3389/fphys.2021.789773

**Published:** 2021-12-23

**Authors:** Oliver Siehler, Shuo Wang, Guy Bloch

**Affiliations:** ^1^Department of Ecology, Evolution and Behavior, Alexander Silberman Institute of Life Sciences, The Hebrew University of Jerusalem, Jerusalem, Israel; ^2^Department of Mechanical and Aerospace Engineering, The University of Texas at Arlington, Arlington, TX, United States; ^3^The Federmann Center for the Study of Rationality, The Hebrew University of Jerusalem, Jerusalem, Israel

**Keywords:** honey bee, social synchronization, circadian rhythm, coupled oscillators, division of labor, substrate borne communication, non-photic entrainment

## Abstract

Honey bees live in colonies containing tens of thousands of workers that coordinate their activities to produce efficient colony-level behavior. In free-foraging colonies, nest bees are entrained to the forager daily phase of activity even when experiencing conflicting light-dark illumination regime, but little is known on the cues mediating this potent social synchronization. We monitored locomotor activity in an array of individually caged bees in which we manipulated the contact with neighbour bees. We used circular statistics and coupling function analyses to estimate the degree of social synchronization. We found that young bees in cages connected to cages housing foragers showed stronger rhythms, better synchronization with each other, higher coupling strength, and a phase more similar to that of the foragers compared to similar bees in unconnected cages. These findings suggest that close distance contacts are sufficient for social synchronization or that cage connection facilitated the propagation of time-giving social cues. Coupling strength was higher for bees placed on the same tray compared with bees at a similar distance but on a different tray, consistent with the hypothesis that substrate borne vibrations mediate phase synchronization. Additional manipulation of the contact between cages showed that social synchronization is better among bees in cages connected with tube with a single mesh partition compared to sealed tubes consistent with the notion that volatile cues act additively to substrate borne vibrations. These findings are consistent with self-organization models for social synchronization of activity rhythms and suggest that the circadian system of honey bees evolved remarkable sensitivity to non-photic, non-thermal, time giving entraining cues enabling them to tightly coordinate their behavior in the dark and constant physical environment of their nests.

## Introduction

Circadian clocks are endogenous pacemakers capable of autonomously generating rhythms of about a day (∼24 h). In order to be ecologically meaningful, these pacemakers need to be synchronized with reliable environmental cycles, a process termed “entrainment” (Dunlap et al., 2004; Helm et al., 2017). Ambient light and temperature cycles, which are influenced by the sun position, are considered the most important environmental cues entraining circadian clocks (known as “time-givers,” or “zeitgebers” in German). The notion that the sun is the pivotal source for entraining cues is consistent with the view that circadian clocks have evolved to adjust the biology of organisms on Earth to predicted daily fluctuations in their environment. Non-photic entrainment has been the subject of less research effort, but yet, there is good evidence that non-photic, non-thermal, cues may act as potent zeitgebers. In recent years, there has been specifically significant progress in research on the mechanisms and entrainment by diet and feeding cycles (e.g., López-Olmeda, 2017; Tomioka and Matsumoto, 2019; Lewis et al., 2020). Additional cues that may entrain circadian rhythms stem from social interactions between individuals of the same species (for a recent review Siehler et al., 2021).

Social entrainment appears to be specifically effective in social animals such as bats and honey bees that live in constant environments in which some or all individuals are not sufficiently exposed to ambient day-night cycles (Favreau et al., 2009; Castillo-Ruiz et al., 2012; Eban-Rothschild and Bloch, 2012; Siehler et al., 2021). In species in which social entrainment is effectual, it was shown to be mediated by diverse cues that are related to the species sensory ecology and social biology, and may include olfactory cues (e.g., rodents and fruit flies), auditory cues (bats and some passerine birds), photic bioluminescence signals (glowworms), or substrate-borne vibrations (honey bees; Siehler et al., 2021).

The Western honey bee *Apis mellifera* provides an excellent model system for research on social entrainment. These highly social insects live in colonies consisted of tens of thousands of individuals that coordinate almost any aspect of their life and nest in dark cavities. As other social insects, they show a division of labor between worker bees performing different activities. In honey bees, the division of labor relates to worker age; young workers initially clean cells inside the nest, later they typically care for (“nurse”) brood which is located in the center of the nest, then they typically perform “middle-age” tasks such as wax comb construction and honey storage, and finally at about 2 to 3 weeks of age, they typically switch to out-of-nest activities such as guarding and foraging (Robinson, 1992). Task performance, however, is plastic and associated with plasticity in circadian rhythms. Nurses and some other nest bees are typically active around the clock with attenuated or no circadian rhythms, whereas foragers have strong circadian rhythms that are necessary for timing visits to flowers, for dance language communication, and for sun compass orientation (Bloch, 2010; Eban-Rothschild and Bloch, 2012). Nevertheless, nurses that are active around the clock in the dark and tightly thermoregulated nest, do have functional pacemakers that measure time and are entrained to the ambient environment (Frisch and Koeniger, 1994; Shemesh et al., 2007, 2010; Rodriguez-Zas et al., 2012; Fuchikawa et al., 2017; Beer et al., 2018). How is the clock of nurses which spend most of their time in the dark and tightly regulated cavity of the nest is synchronized with ambient day-night cycles? There is good evidence suggesting that the clock of nurse bees is socially entrained by foragers who are directly exposed to the outside environment (Bloch et al., 2013; Siehler et al., 2021).

Worker bees are very sensitive to social time-givers and exposure to the hive environment only during the first 2 days (but not only 1 day) post pupa eclosion is sufficient for stable entrainment to the colony phase (Fuchikawa et al., 2016). We recently showed that social synchronization is effective even among newly emerged bees that were each caged individually in an array of connected cages placed on the same substrate. Given that such young bees typically show weak circadian rhythms in locomotor activity, at best, their effective synchronization to a common phase indicate that their circadian system is remarkably sensitive to social time-giving cues (Siehler et al., 2021). Entrainment to the colony phase is not compromised in young bees that are caged in the hive centre that is constantly dark and tightly thermo-regulated (Eban-Rothschild et al., 2012; Fuchikawa et al., 2016). These observations suggest that entrainment does not require sampling the environment at the hive entrance or the hive periphery in which temperature may vary substantially over the day (Giannoni-Guzmán et al., 2021). Taken together, the studies on social synchronization of activity rhythms in honey bees fit best with self-organisation models in which the sum activity of the bees in a group generates fluctuations in the microenvironment of the hive. These activity related oscillations in turn, entrain the circadian clocks of an increasing number of bees; the more individuals are entrained to a similar phase, the resonance of their common phase more effectively entrains the clock of bees with a different phase (Bloch et al., 2013; Siehler et al., 2021). The agreement with self-organisation models further suggests that surrogates of worker activity mediate social synchronization in honey bee colonies, and focuses the research on proxies of activity that can entrain the circadian clocks of honey bees.

In typical colonies, the main factor determining the common phase is apparently forager activity that is entrained by ambient time-givers experienced when foraging outside the nest (Beer et al., 2016; Fuchikawa et al., 2016). Bees are entrained to the colony phase even if separated from the rest of the colony by double-mesh dividers indicating that direct contact with other bees is not necessary for social entrainment in honey bees (Beer et al., 2016; Fuchikawa et al., 2016). Indeed, volatiles drawn from a free-foraging colony, but not from a similarly heated but unpopulated hive, stably entrain circadian rhythms in locomotor activity in groups of young honey bees (Siehler and Bloch, 2020). These observations are consistent with earlier lab studies showing that allowing airflow between two groups of bees separated by an impermeable partition improved their synchronization to a common daily rhythm (Moritz and Kryger, 1994). There is also good evidence that substrate-borne vibrations generated by forager activity act as potent social time-giver entraining circadian rhythms in honey bees (Siehler and Bloch, 2020; Siehler et al., 2021).

Here, we tested the hypothesis that close distance contact between foragers and young bees contributes to social synchronization of circadian rhythms in locomotor activity. We further hypothesized that foragers socially synchronize young bees and not vice-versa, because young worker bees typically have weaker rhythms compared to foragers and other older workers. To address these hypotheses we individually isolated bees, each in a small cage, in a tightly regulated lab environment, and manipulated the contact between foragers and callow bees. Adjacent cages were connected with small tubes with a mesh separation that prevented moving from one cage to the other. Tube connection may also improve the propagation of volatiles and substrate-borne vibration that can entrain circadian rhythms in young bees (Siehler et al., 2021; see above). To determine coupling strength between each pair of bees we used our recently developed ICON pipeline that efficiently and reliably infers the dynamics of even complex network of coupled oscillators and can be used with noisy data such as locomotor activity (Wang et al., 2018). We predicted that if cage contact improves social synchronization, then phase coherence in the circular statistics and coupling strength in the ICON analyses (see Methods below) will be higher for bees in connected compared to unconnected cages. Taking into account previous evidence that substrate-borne vibrations can mediate social synchronization (Siehler and Bloch, 2020; Siehler et al., 2021), we also predicted that coupling strength is higher for bees placed on the same tray compared to bees at a similar distance but on a different tray.

## Materials and Methods

### Honey Bees

We maintain honey colonies according to standard beekeeping techniques at the Bee Research Facility at the Edmond J. Safra campus of the Hebrew University of Jerusalem, Givat Ram, Jerusalem, Israel. To obtain newly emerged bees, we removed honeycomb frames with emerging worker pupae, brushed off all adult bees, and with no delay, transferred each frame into a separate lightproof container. We placed the frames in an incubator (33 ± 1^°^C, 60% ± 5%RH) for the bees to emerge. We collected the emerging bees within 24 h post emergence under dim red light (DD; using Edison Federal EFEE 1AE1 Deep Red LED; mean wavelength = 660 nm, maximum and minimum wavelengths = 670 and 650, respectively) to avoid influences of light on the circadian system (e.g., photic entrainment, aftereffect).

### Monitoring Locomotor Activity

We placed each bee individually in a monitoring cage made of a modified Petri dish (diameter = 90 mm) provisioned with *ad libitum* sugar syrup (50% w/w) and pollen. The monitoring cages with the bees were placed in a tightly regulated environmental chamber (29 ± 1°C, 60 ± 5%RH). The chamber was illuminated with dim red light (Edison Federal EFEF 1AE1 Far (Cherry) Red LED; mean wavelength = 740 nm, maximum and minimum wavelengths were 750 and 730, respectively). Locomotor activity (measured as number of pixels traveled over a time unit on the camera field of view) was recorded automatically at a frequency of 1 Hz with the ClockLab data acquisition system (Actimetrics Inc., Evanston, IL, United States). The system is composed of four infrared light-sensitive black and white cameras (Panasonic WV-BP334, 0.08 lux CCD video cameras in the 1^st^ experiment; Sentech STC-MB33USB mini-USB video cameras with Computer TZ32910CS-IR lenses in the 2^nd^ experiment) and a high-quality monochrome image acquisition board (National Instruments IMAQ 1409, or USB-6501 interface in the 1^st^, and 2^nd^ experiments, respectively). Each camera covers a single tray on which we placed 30 cages. Using this system, we could monitor up to 116 bees in each trial. The remaining four cages, one on each tray, were left vacant and used as a control recording background noise.

### Analyses of Circadian Rhythms

**FIGURE 1:**
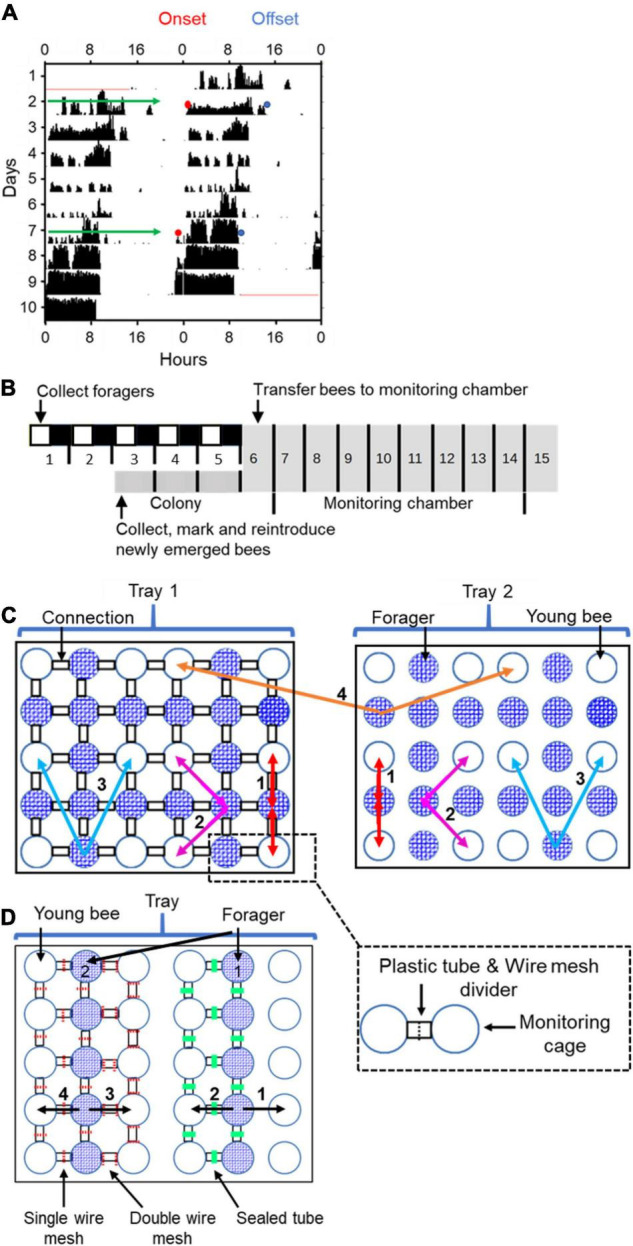
Experimental and methodological details. **(A)** Double-plotted actogram of a representative individually isolated worker bee. The *y*-axis shows days in the monitoring cage, and the *x*-axis shows the time of day, double plotted for easier visual detection of rhythms. The height of the black bars within each day corresponds to the distance moved in pixels in a 10 min. bin. The blue and the red dots in days 2 and 7 show the estimated times for the onset and offset of activity, respectively. **(B)** Experimental outline for the experiment testing the influence of close distance contact on social synchronization between young bees and foragers. The numbers correspond to the days of the experiment. The horizontal bar shows the illumination regime that the bees experienced: filled box – dark, white boxes – light, grey boxes – constant darkness. The light- dark illumination regime during the first 5 days is 5 h advanced relative to the time of sunrise. During this entrainment session, the foragers were placed in an environmental chamber (30 ± 1°C, 55% ± 5%RH). On day 6 of the experiment, each forager or young bee was transferred to the locomotor activity monitoring chamber and placed in an individual cage. **(C)** A schematic illustration of cage arrangement in Experiment 1 showing two of the four trays. Each tray houses 30 cages that are constantly recorded by a video camera. The cages are either connected (left tray; the black rectangles denote transparent connecting tubes installed with a wire mesh divider, depicted by a dashed line in the inset) or not (right tray). Circles with blue mesh filling and open circles depict monitoring cages housing either foragers or young bees, respectively. The coloured arrows and numbers depict the various types of coupling strength analyses: (1) Bees with and without direct contact with their neighbour bees; (2) 2^nd^ order neighbours. Connected and unconnected cages one step away from the direct neighbours; (3) 3^rd^ order neighbours. As in (2), but one step further away; and (4) The coupling strength for unconnected bees to others on the same or on a different tray but located at a similar distance. In groups (1) and (2) we compared the coupling in both directions (Forager → Young bee as well as Young bee → Forager). In groups (3) and (4) we only compared the direction Forager → Young bee. **(D)** A schematic illustration of cage arrangement on a tray in Experiment 2. The numbered vectors depict the analysis we performed: (1) Neighbour bees with no cage connection. (2) Neighbour bees in cages connected with a sealed tube. (3) Neighbor bees in cages connected with a tube separated with a double 8’ wire mesh divider. (4) Neighbour bees in cages connected with a tube separated with a single 8’ wire mesh divider. We designate the foragers for analyses 1 and 2 as forager 1 and for analyses 3 and 4 as forager 2. Other details as in panel **(C)**.

We used the ClockLab software package (Actimetrics, United States) for the analyses of circadian rhythms. We used the χ2 periodogram analysis with 10 min bins to determine whether the activity rhythms of a given bee are statistically significant. As a proxy for the strength of circadian rhythms we used the *“Power”* which we calculate as the height of the periodogram plot peak above the α = 0.01 significance *p*-value threshold line (for more details see Klarsfeld et al., 2003; Yerushalmi et al., 2006). As indices for the phase, we recorded for each day the time of onset and offset of the daily bout of activity (honey bees are diurnal and typically show higher levels of activity during the day or subjective day). The precise time of the onset or offset was defined as at least three consecutive 10-min bins each with activity reaching at least 10% of the maximum activity per bin during this day separated by a period of at least 5 h of reduced activity between the offset and the following onset (see Figure 1; following Fuchikawa et al., 2016). We used for the analyses only bees with statistically significant circadian rhythms (χ^2^ periodogram analysis, *p* < 0.01; with a major period peak between 20 and 28 h) for which we could unambiguously determine the onset/offset of activity, and omitted those for which the periodogram was below our statistical threshold.

We used the Oriana circular statistics software package (KCS, United States) to determine the degree of synchronization and the phase coherence among bees within each treatment group. For these analyses we used data of the bees for which we could unambiguously determine the phase. For all circular statistics analyses we used the onset, offset, and the median between these two indices as indices for phase. Given that for more bees we could unambiguously determine the onset than the offset, and that the analyses using the three phase indices were overall similar, we chose to present the onset data for which the statistical power was stronger. We used the Rayleigh test to determine if phase synchronization among a group of bees is significantly different from random distribution. The mean length of the Rayleigh vector was used as an index for the degree of synchronization.

### Estimating the Coupling Function Between Bees

We used our customized data-driven graph-theoretic approach (ICON; Wang et al., 2018) to quantitatively describe the coupling function between each pair of bees. The pipeline we used includes wide bandpass basic filter, the ICON calculation, and connectivity analysis (see below), allowing us to efficiently and reliably infer the dynamic connectivity of oscillators from noisy measurements and is therefore appropriate for the locomotor activity data of honey bees. We modeled the honey bee locomotor activity data as a dynamic feature reflecting this complex dynamic network of honey bees with interactions, where the dynamics of each honey bee consists of its own rhythm and the influence from other honey bees. In particular, we consider the broadly defined complex network constituted by a population of *N* interacting honey bees (i.e., oscillators with a period of 20–28 h). The time-evolution of such a network follows the dynamic law governed by the rhythm of honey bees *f*(*x*_*i*_) (i.e., oscillator’s self-dynamics) and the influence by other honey bees *K_ij_*(*x*_*i*_,*x*_*j*_), given by


(1) $${\dot{x}}_{i}\,\left(t\right)=f\,\left(x_{i}\right)\,\sum_{\begin{array}{c} j=1\\ j\,i \end{array}}^{N}K_{i\,j}\,\left(x_{i},x_{j}\right);i=1,\ldots ,N,$$

where *x*_*i*_(*t*) is the locomotor activity of the *i^th^* honey bee at time *t*, the function *f*(*x*_*i*_) represents its baseline dynamics, such as its natural frequency, and*K_ij_*, *i*,*j* = 1,…,*N*, is the coupling impact from the *j^th^* honey bee to the *i^th^*.

We first approximate the natural and coupling dynamics, *f* and *K*_*ij*_ in (1), respectively, using complete orthonormal bases. Based on Kuramoto’s model (Kuramoto, 1984), we choose Fourier base function with periods ranging from 16 to 32 h for our weakly coupled oscillatory honey bee network because *f* and *K*_*ij*_ should be periodic functions (which simplifies the formulation of the following linear inverse problem, for more details see Wang et al., 2018). We next formulated this complex non-linear estimation as a typical large-scale linear inverse problem for each honeybee


(2) $$\min_{z^{\left(i\right)}}{||y^{\left(i\right)}-A^{\left(i\right)}\,z^{\left(i\right)}||}_{2}$$

where *y*^(*i*)^ ∈ ℝ^(*M*−1)^ is the data vector whose elements $y_{j}^{\left(i\right)}=\frac{\Delta \,{\tilde{x}}_{j}^{\left(i\right)}}{\Delta \,t_{j}}$, *j* = 1,…,*M*−1, denote the state difference with Δ*t*_*j*_ = *t*_*j* + 1_−*t*_*j*_ being the data sampling time interval; *A*^(*i*)^ ∈ ℝ^(*M*−1) = (2*rN* + 1)^ is the matrix involving orthonormal bases and *z*^(*i*)^ is the coefficient vector to be estimated which includes the connectivity information. The detailed formulation of Eq. 2 as well the mathematical validation of the ICON procedure, including reliability and efficiency with respect to variations in network size, connectivity rate, coupling strength, and noise intensity ratios, etc., which were conducted through numerical simulations and statistical hypothesis tests are detailed in the main text and the Supplementary Information of Wang et al. (2018).

A basic step for solving this large-scale linear inverse problem is to compute the Moore-Penrose pseudoinverse using the singular value decomposition (SVD). In this work, we first compared the computational efficiency, accuracy, and time complexity of ICON through numerical experiments for synthetic networks in different sizes and sparsity levels using existing methods such as SVD, iterative method with thresholding, and Bayesian learning technique (SI, Wang et al., 2018). We chose to implement the truncated singular value decomposition SVD (TSVD) method, which is more efficient compared to the standard SVD method because it only focuses on the most significant singular values that determine the linear inverse. Then, we can quantitatively measure the coupling strength from the *j*^th^ honey bee to the *i^th^* (i.e., the magnitude of the function *K*_ij_) using the corresponding coefficients as in the solution *z*^(*i*)^. For the figure presentations we normalized the data such that the maximal value was converted to 1 and the value of each measure was calculated as the propostion of this maximun giving a value ranging between 0 and 1.

### Experiment 1. The Influence of Direct Contact on Social Synchronization

We tested the hypothesis that close distance contact (hereafter refer to as “direct contact”) between foragers (strong oscillators) and young (callow) bees (weak oscillators) facilitates social synchronization among worker bees. We also hypothesized that the foragers synchronize the rhythms of the callow bees and not vice versa. To test these hypotheses, we analysed circadian rhythms in locomotor activity of individually isolated bees in a constant lab environment. In the treatment group, bees were placed in adjacent Petri dishes that are connected with transparent plastic tubes (length ∼1.5 cm; inner diameter = 1 cm). The tubes were divided by an 8′ wire mesh divider, such that they could smell, touch, antennate, and lick each other, but could not move from one cage to the other (Figure 1C). Control bees were placed in similar cages, but could not contact their neighbours because the Petri dishes were not connected with a tube.

The experiment outline is summarised in Figure 1B: On day 1 of the experiment, we collected foragers and transferred them to wooden lab-cages (∼25 bees per cage). Each cage was provisioned with *ad-libitum* sugar syrup and pollen and was housed in an environmental chamber (30 ± 1°C, 55% ± 5%RH). During the first 5 days, the foragers experienced a 12 h light: 12 h dark (12:12 LD) illumination regime, that was 5 h advanced relative to time of sunrise. On Day-3, we collected newly emerged bees, marked each with a paint dot and reintroduced them back into their mother colony. The marked bees were kept in the colony for 2 days, a period that was previously shown to be sufficient for stable entrainment to the colony phase (Fuchikawa et al., 2016). On Day-6, we collected the marked young bees from the hive and the foragers from the lab, and transferred them to individual cages in the locomotor activity monitoring chamber in which their activity was monitored for at least seven successive days. We assumed that the young bees were entrained to the colony phase, and the foragers to the phase of the LD cycle that is 5 h advanced relative to the colony phase. At the end of the experiment, we calculated the percentage of bees that survived until the end of the experiment and used Pearson-Chi square tests to assess the effect of cage connection on survival. We used one-way ANOVA to compare the strength (Power of rhythmicity) of circadian rhythms. Next, we determined the time of onset and offset of the daily bouts of activity on monitoring days 2 and 7 (Figure 1A). We used these data to compare the degree of synchronization and the phase for each group of bees. We used the Rayleigh vector to calculate the average time of onset of morning activity on days 2 and 7, and paired *t*-tests for determining if the shift between these 2 days is statistically significant. We performed one-way ANOVA test within each trial to compare the effect of treatment on the difference between the time of onset on days 2 and 7. We used the ICON pipeline (Wang et al., 2018) to quantitatively describe the coupling function between each pair of bees and unpaired *t*-tests to compare coupling strength of bees subjected to different treatments.

The arrangement of cages with foragers and young bees on the trays is shown in Figure 1C. Control trays were arranged in a similar way, but with the Petri-dishes not connected to each other. We predicted that if direct contact between foragers and young bees improves social synchronization, then the bees in connected cages should be better synchronized with each other, and have stronger coupling than similar bees in unconnected cages. Furthermore, the phase of connected bees is predicted to be more similar compared to bees on cages that are not connected. We also predicted that the coupling strength of forager → callow will be higher compared to callow → forager.

### Experiment 2. The Influence of the Type of Partition Between Adjacent Cages on Social Synchronization

Given that in Exp. 1 synchronization was improved by direct contact, we performed an additional experiment in which we manipulated the propagation of information between the cages. Adjacent cages were either not connected (1 in Figure 1D), or connected with similar transparent plastic tubes to these used in the first experiment. The tubes were equipped with one of the following dividers (“Treatments”): (a) A single 8′ wire mesh partition (SM; as in the first experiment), enabling direct contact and spread of volatiles and vibrations; (b) a double 8′ wire mesh partition (DM), enabling the spread of volatiles and vibrations, but preventing direct contact; and (c) the tube was sealed with hot glue (S), enabling the spread of vibrations but not volatiles or direct contact (#2 in Figure 1D). The bees in this experiment were handled and entrained as in Experiment 1. The foragers experienced a 12 h light: 12 h dark (12:12 LD) illumination regime, that was 5 h advanced relative to time of sunrise. Newly emerged bees were paint marked and reintroduced back into their mother colony in which they were entrained to the colony environment for 2 days (Fuchikawa et al., 2016). After 6 days, we collected the bees from both groups and transferred each one of them into an individual cage in the locomotor activity monitoring system. Their locomotor activity was monitored for at least seven successive days. At the end of the monitoring sessions, we performed similar analyses to these detailed for Exp. 1. In addition to the one-way ANOVA tests for each trial (as in Exp. 1), we also performed two-way ANOVA for the two trials pooled together, with treatment (type of cage connection) and the trial as factors.

## Results

### Experiment 1. The Influence of Direct Contact on Social Synchronization

**FIGURE 2:**
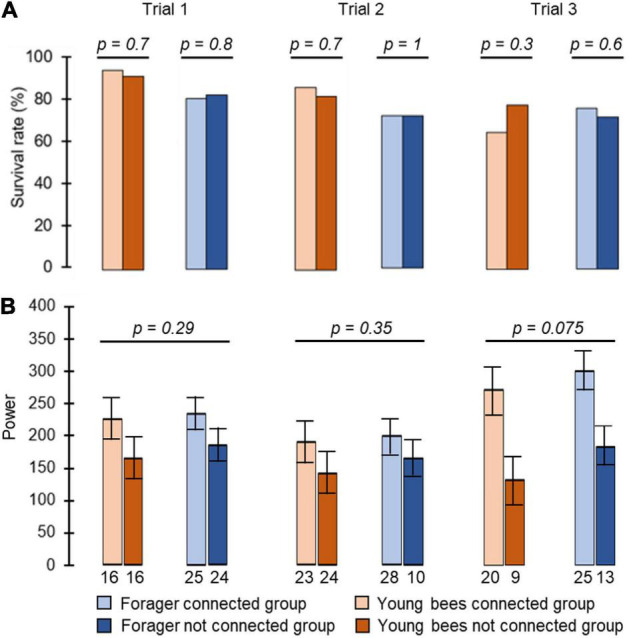
The influence of cage connection and worker type on the strength of circadian rhythms and survival rate. **(A)** Percentage of bees that survived until the end (day 8 in monitoring chamber). The *p*-values above the plots summarise the results of Pearson Chi square tests. **(B)** The strength of circadian rhythms in locomotor activity. We included arrhythmic bees and assigned them a value of Power = 0. The *p*-values above plots summarise the results of one-way ANOVA test. The bars show mean and the whiskers show ± SE. Each column summarizes a different trial.

We monitored locomotor activity for 280 bees: 87, 77, and 116 bees, in Trial 1, 2, and 3, respectively. Cage connection did not influence survival rate for either foragers or young bees (foragers unconnected = 83%, 72%, 72%, connected = 81%, 72%, 76%, young bees unconnected = 92%, 82%, 78%, connected = 95%, 86%, 65% for trials 1, 2, and 3, respectively; Pearson Chi square test: foragers, *p* = 0.8, *p* = 1, *p* = 0.6; young bees: *p* = 0.7, *p* = 0.7, *p* = 0.3 for trials 1, 2, and 3, respectively; Figure 2A). Cage connection also did not affect the strength of circadian rhythms with a mean Power of 236.5 ± 11 SE; 200 ± 23 SE; 300 ± 43 SE for trials 1, 2 and 3, respectively, for the *Connected Foragers*; 184 ± 11 SE; 165 ± 33 SE; 183 ± 42 SE for the *Unconnected Foragers*; 225 ± 12 SE; 189 ± 26 SE; 272 ± 43 SE for the *Connected Young bee* and 165 ± 13 SE; 143 ± 24 SE; 131 ± 46 SE for the *Unconnected Young bee* (one-way ANOVA test; see details in Figure 2B).

**FIGURE 3:**
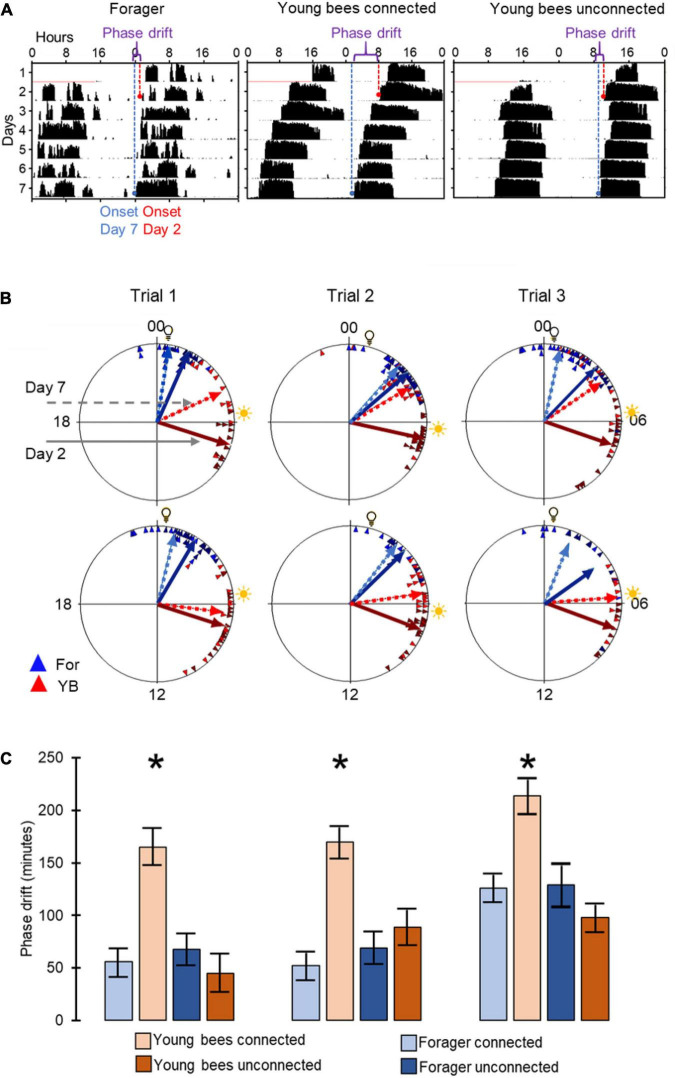
Phase synchronization of young bees and foragers housed in cages with or without connection to adjacent cages. **(A)** Representative actograms showing the phase difference between Day-2 (red) and Day-7 (blue). For details of actogram see Figure 1A. **(B)** Circular plots summarizing phase synchronization in days 2 and 7. Upper row – bees housed in cages connected to each other; lower row – bees in similar cages but not connected to each other. The time of day is depicted on the plot perimeter. Light bulb icon depict the time of light on during the forager entrainment session, which was 5 h advanced relative to the time of sunrise (sun icon). Each triangle depicts the onset of the morning bout of activity for an individual bee. The vectors point to the average onset time, and their length corresponds to the degree of phase coherence. Foragers (For) are depicted in blue and young bees (YB) in red. Solid and dashed lines - mean vector calculated on days 2 and 7 of the experiment, respectively. **(C)** Phase difference between the onset of activity on days 2 and 7. Asterisks depict a significant difference in a paired *t*-test comparing the time of onset of activity on Day 2 vs. Day 7. Sample sizes (number of bees for which we could calculate the onset of the morning bout of locomotor activity): For bees housed in connected cages: Foragers: 18, 22, 20; Young bees: 12, 22, 16 in Trial 1, 2, and 3, respectively; for bees housed in unconnected cages: Foragers: 19, 10, 9; young bees: 14, 19, 8, respectively.

To assess social influences on the phase of circadian rhythms we first used circular statistics. All groups of bees showed significant synchronization of circadian rhythms in locomotor activity when tested on both day 2 and day 7 of the experiment (Rayleigh vector with *p*<<0.05). We found that after 7 days, the phase difference between young bees and foragers was smaller for the connected compared to the unconnected bees (Figures 3A,B). In all three trials, the phase difference between the 2^nd^ and 7^th^ day was statistically significant (paired *t*-test) only for the young bees in cages connected to adjacent cages with foragers (Figure 3C). This finding is consistent with the premise that cage connection improves the synchronization of young bees to the phase of foragers.

**FIGURE 4:**
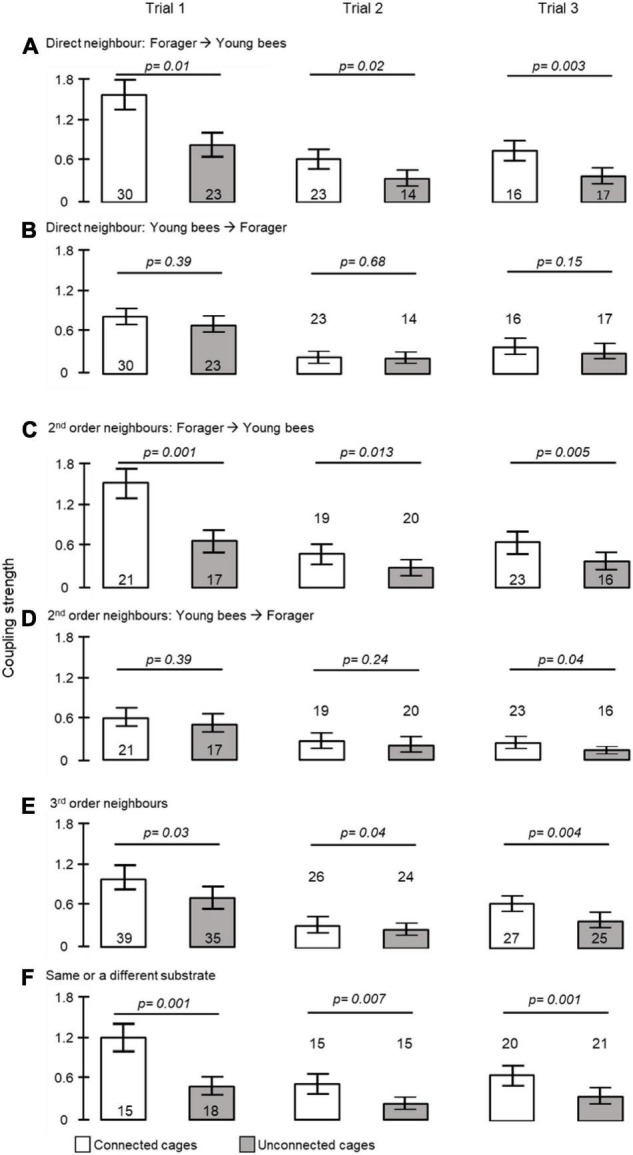
The influence of direct contact, distance and being placed on the same substrate on coupling strength. **(A)** Foragers to callows in adjacent cages; **(B)** callows to foragers in adjacent cages; **(C)** foragers to 2^nd^ order callow neighbours; **(D)** callows to 2^nd^ order forager neighbours; **(E)** foragers to 3^rd^ order neighbours; **(F)** bees in unconnected cages at a similar distance but placed on either the same or on a different tray (see Figure 1C for details of tray organization and type of analyses). The bars show mean ± SE, sample size is shown within or above bars. The *p*-values above the bars summarize the results of unpaired *t*- tests.

To further assess social influences on circadian rhythms in locomotor activity we estimated the coupling strength for pairs of bees housed in either connected or unconnected cages (Figure 1C). The coupling strength of foragers to young bees was stronger when housed in connected compared to unconnected cages in all three trials (unpaired *t*- test, *p* = 0.01, *p* = 0.02, *p* = 0.003, in trials 1, 2, and 3, respectively, Figure 4A). Cage connection did not influence coupling strength in the opposite direction of young bees on neighbor foragers (*p* = 0.39, *p* = 0.68, *p* = 0.15; Figure 4B). Cage connection improved synchronization also among foragers to 2^nd^ order young neighbours (#2 in Figure 1C) that are further apart (*p* = 0.001, *p* = 0.013, *p* = 0.005; Figure 4C), but not at the opposite direction, callow → foragers (*p* = 0.39, *p* = 0.24, *p* = 0.04; Figure 4D). The effect of cage connection was significant also when we compared 3^rd^ order neighbours (#3 in Figure 1C). Coupling strength was stronger for foragers → young bees in connected compared to unconnected cages (*p* = 0.03, *p* = 0.04, *p* = 0.004, for trials 1–3, respectively; Figure 4E). Finally, we compared the coupling strength among bees in unconnected cages on the same or on a different tray, but at a similar distance (#4 in Figure 1C), and found stronger coupling for the bees on the same tray (*p* = 0.001; *p* = 0.007; *p* = 0.001, respectively; Figure 4F).

### Experiment 2. The Influence of the Type of Partition Between Adjacent Cages on Social Synchronization

**FIGURE 5:**
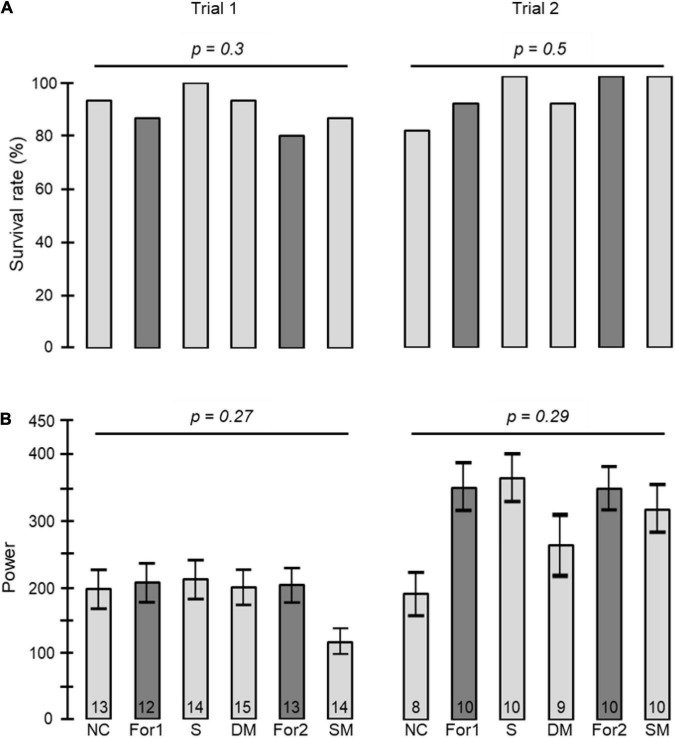
The influence of the type of cage connection on the power of circadian rhythms and survival rate. **(A)** Percentage of bees surviving until the end of the monitoring session. The *p*-values summarise the results of Pearson Chi square tests with Fisher’s exact tests on contingency table). **(B)** The power of circadian rhythms in locomotor activity. The bars show mean ± SE, sample size is shown within bars. The *p*-values above the plots summarize the results of one-way ANOVA tests. NC – young bees not connected, For1 – Foragers placed in raw 1, For2 – Foragers placed in raw 2 (see Figure 1D for the tray location of foragers from groups For1 and For2), S – young bees connected with a sealed tube, DM – young bees connected with a double-mesh divider, SM – young bees connected with a single-mesh divider. For more details on the types of cage connection, see Figure 1D.

We included in our analyses 138 bees for which we could reliably determine the daily onset of the morning activity bout (Trial 1, *n* = 81; Trial 2, *n* = 57 bees). Survival rate was good and similar for foragers and young bees (80–100% for both groups) and was not affected by the type of connection between adjacent cages (Pearson Chi square test with contingency table and Fisher’s exact tests for comparing the different manipulations; *p* = 0.3 and *p* = 0.5 in trials 1 and 2, respectively; Figure 5A). The type of connection between the cages (i.e., treatment) did not influence the strength of circadian rhythms (ANOVA test *p* = 0.27, *p* = 0.29, in trials 1 and 2, respectively; Figure 5B).

**FIGURE 6:**
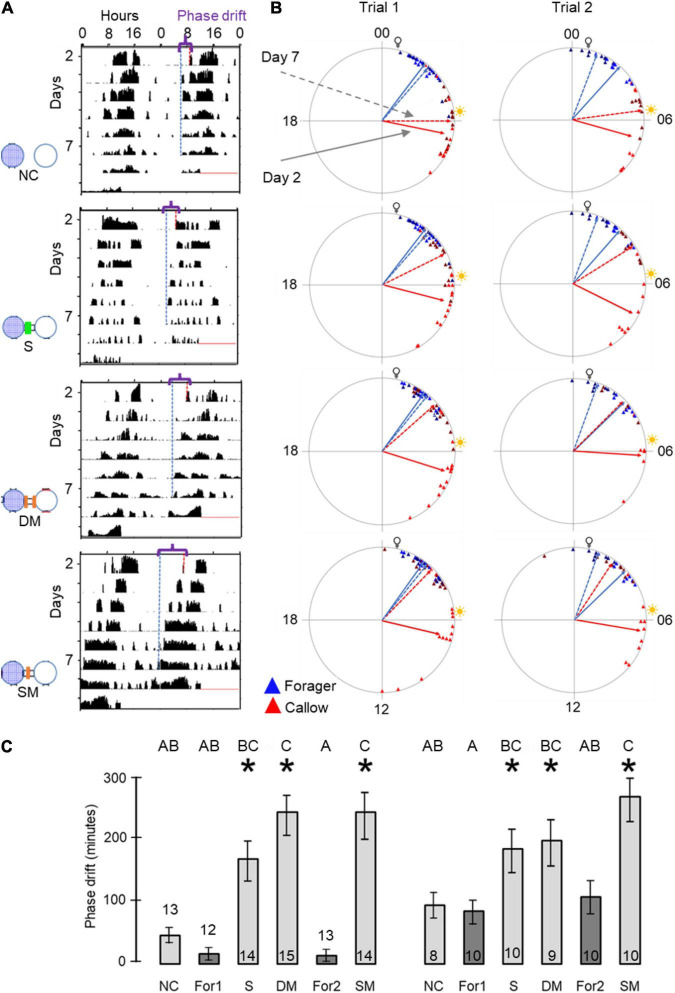
The influence of the type of cage connection on the synchronization of young bees and foragers. Each row in **(A,B)** presents a different treatment. From top to bottom: Not connected (NC), connected with a sealed tube (S), connected with a tube with double mesh partition (DM), connected with a tube with a single mesh partition (SM). Sample sizes as detailed in panel **(C)**. **(A)** Representative actograms showing the difference (marked with a curly bracket ({) between the onset of activity on days 2 and 7 (red and blue dash lines, respectively) for callow bees subjected to the different treatments. For details of actogram see Figure 1A. **(B)** Circular plots summarizing phase synchronization in days 2 and 7. The two columns depict the results of the two trials. Details of circular plots as in Figure 3B. **(C)**. Phase difference between the onset of activity on days 2 and 7. Asterisks depict a significant difference in a paired *t*-test comparing the onset of the morning bout of activity on days 2 and 7. Treatments marked with different letters are statistically different in a one-way ANOVA test followed by Tukey HSD *post hoc* comparison.

Our circular statistics analyses suggest that at day 7 of the experiment, the phase difference between young bees and foragers is larger (i.e., less similar) for bees in unconnected cages compared to the other three treatments in which the cages were connected with a tube (Figure 6). These findings suggest that the young bees in cages connected to cages housing foragers shifted their phase toward that of the foragers. In order to more quantitatively assess the foragers influence, we compared the phase difference between days 2 and 7 for bees subjected to the different treatment. In both trials, the phase significantly changed between days 2 and 7 (paired *t*-test, *p* < 0.05; Figure 6C) in the *sealed tube, double-mesh, and single-mesh divider group*, but not for the unconnected bees or the foragers in the two different locations of the tray (For1 and For2). In order to compare bees subjected to different treatments, we first performed one-way ANOVA tests for each trial separately. The summary of these analyses are shown in Figure 6C. Although the trends were similar in the two trails, some of the differences did not cross the statistical significant threshold in the second trial. To increase our statistical power, we further performed a two-way ANOVA for the two trials together. This analysis revealed a significant effect for treatment but not for the trial or interaction (Table 1). Complementary Tukey HSD *post hoc* paired comparisons showed that the shift in the time of onset was significantly larger for the young bees in the single mesh (SM) and double mesh (DM) treatments compared to the two groups of foragers (F1 and F2) or the unconnected young bees (NC). The bees in the sealed tube connection treatment (S) showed a larger shift compared to the two groups of foragers, but not compared to the NC treatment. The shift was larger for the SM compared to the S but not to the DM treatment (Table 1). These analyses suggest that the single-and double-mesh partitions enable the young bees to shift their phase of activity toward that of their neighbor foragers. The sealed tube partition apparently attenuated but not blocked this effect.

**TABLE 1:** A summary of a two – way ANOVA with treatment and trial as factors for the difference between the onset of the morning bout of activity on days 2 and 7 for bees in cages with different type of connections.

(A) ANOVA summary table					
**Source**	**DF**	**Sum of square**	**Mean square**	***F***	**Pr > F**
Group	5	528032.533	105606.507	14.606	0.000
Trial	1	11531.913	11531.913	1.595	0.209
Group × Trial	5	41558.905	8311.781	1.150	0.338

**(B) Summary of *post hoc* analyses**					
**Treatment**	**Mean estimator**	**Groups**			

SM	245.921	A			
DM	210.952	A	B		
S	167.976		B	C	
NC	110.500			C	D
F2	79.542				D
F1	71.154				D

*Treatments with different letters are statistically different in Tukey HSD post hoc pair comparison. For details of treatments see Figure 1D.*

**FIGURE 7:**
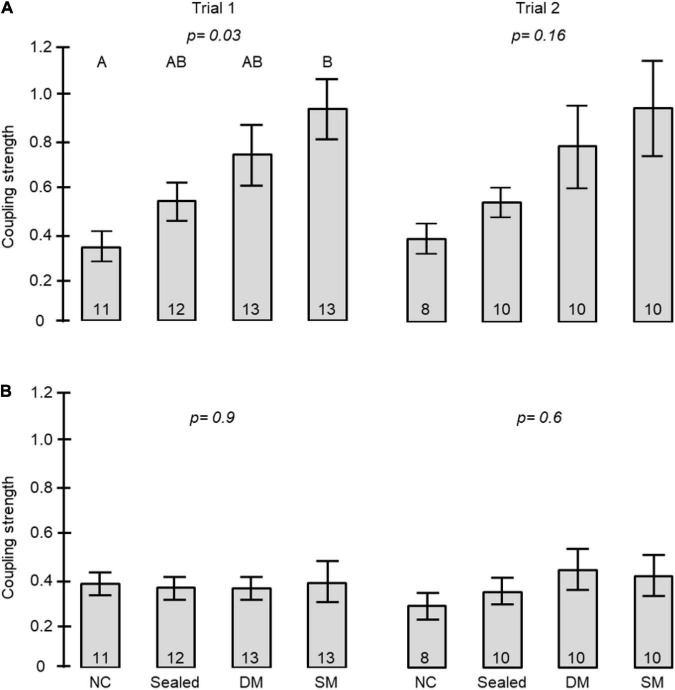
The influence of the type of connection between adjacent cages on the coupling strength between foragers and young bees in adjacent cages. The bars show mean ± SE, sample size is shown within bars. The *p*-values above the bars summarize the results of one-way ANOVA. **(A)** The coupling strength of Foragers to Young bees. Bars with different small letters are different in Tukey *post hoc* comparison. Table 2 summarises a Two-Way ANOVA for the two trials together. **(B)** The coupling strength of Young bees to Foragers. For more details on the types of connections see Figures 1D, 5, 6.

The coupling strength of foragers to callow bees in neighbor cages was influenced by the type of connection between the cages (i.e., treatment) in Trial 1 (one-way ANOVA test *p*-value = 0.03). *Post hoc* comparisons revealed a significant difference between the coupling strength of bees housed in cages with no connection and those in cages connected with a tube equipped with a single mesh divider (Tukey *post hoc* tests *p* = 0.025). The trend was overall similar in the 2^nd^ trial, but the differences were not statistically significant (*p*-value = 0.16). However, the effect sizes showed a similar trend to that obtained in the first trial (Figure 7A). Young bees with no connection to foragers (NC) showed the weakest, and those divided by a single wire mesh (SM) showed the strongest, coupling strength. Bees connected with a sealed tube or a double-wire mesh showed intermediate values. A two -way ANOVA for the pooled data of the two trials revealed a significant effect of Treatment but not Trial or their interaction (Table 2A). Complementary *post hoc* analyses showed statistically significant differences between the SM and NC groups, but not for any of the other comparisons. The coupling strength was much lower in the opposite direction: Young bees → Foragers (Figure 7B) and with no effect for the type of connection between the cages.

**TABLE 2:** A summary of a two – way ANOVA with Treatment and Trial as factors for the coupling strength of bees in cages with different type of connections.

(A) An ANOVA summary table					
**Source**	**DF**	**Sum of square**	**Mean square**	***F***	**Pr > F**
Treatment	3	3.997	1.332	4.939	**0.003**
Trial	1	0.012	0.012	0.045	0.832
Treatment × Trial	3	0.007	0.002	0.009	0.999

**(B) Summary of *post hoc* comparisons**					
**Treatment**	**Mean estimator**		**Groups**		

SM	0.905	A			
DM	0.726	A		B	
S	0.507	A		B	
NC	0.331			B	

*Treatments with different letters are statistically different in Tukey HSD post hoc pair comparison. For details of treatments see Figure 1D. Bold values are standard statistical terms.*

## Discussion

We combined a tightly controlled lab assay and a new pipeline to assess social synchronization of activity rhythms among honey bee foragers and young bees, each isolated in a separate monitoring cage. This system enables precise manipulation of the information transferred between cages and fine-grained analyses of social influences on activity rhythms. Our findings support and extend evidence that honey bee foragers (“strong oscillators”) can effectively synchronize the daily activity rhythms of young bees (“weak oscillators”). We further show that young bees have little influence, if at all, on the circadian phase of forager activity. The forager effect was stronger when their cages were connected with small tubes separated by a single mesh partition. The influence of the foragers was not limited to callows in adjacent cages, but was also significant when assessing 2^nd^ and even 3^rd^ order neighbours. Given that in each trial we had only a few dozen foragers in an entire environmentally regulated chamber, it is not likely that the foragers achieved this effect by regulating the environment of the whole chamber. Rather, our findings are consistent with the premise that the tube connection improves social synchronization, or that the cage connections facilitated the spread of time-giving social cues. Our results are based on two or three trials, each with bees from a different source colony and a robust data set (more than 250 and 130 individually monitored worker bees in the 1^st^ and the 2^nd^ experiments, respectively). Given that bees in each colony are the offspring of different queens and drones, our findings are not limited to certain genotypes or laboratory lines.

To better understand the nature of the cues synchronizing bees in the array of connected cages, we further manipulated the type of partition in the tubes connecting adjacent cages. The finding that the phase of young bees moved toward that of the foragers even when separated with sealed tubes or with tubes with a double mesh partition is consistent with earlier evidence that bees can socially synchronize their activity rhythms without direct contact (Moritz and Kryger, 1994; Beer et al., 2016; Fuchikawa et al., 2016; Siehler and Bloch, 2020; Siehler et al., 2021). The clear, but statistically not significant, trend toward better synchronization of bees in cages connected with sealed tubes (Figures 6, 7; Tables 1, 2) is consistent with the hypothesis that cage connection improved social synchronization because it facilitates the propagation of substrate vibrations. The potency of substrate borne vibrations was also established in Exp. 1 showing that bees on the same tray have stronger coupling strength compared to bees at a similar distance but on a different trey (Figure 4F). These results extend and support earlier studies emphasizing the importance of substrate borne vibrations for social synchronization in honey bees (Siehler and Bloch, 2020; Siehler et al., 2021) and is consistent with evidence that low frequency vibrations (of about 10–40 Hz) affect the levels of activity in honey bees and can be used as communication signals (Gahl, 1975; Schneider and Lewis, 2004; Stefanec et al., 2021). Vibrations are also known to convey information in additional social insects (reviewed by Hunt and Richard, 2013) and can entrain circadian rhythms of locomotor activity in the solitary to facultatively gregarious fruit flies (Simoni et al., 2014). Nevertheless, the phase drift towards the foragers in Exp. 2 was smaller for bees in cages connected with sealed tubes compared to those connected with tubes with a single mesh partition (Figure 6C; Table 1) and a clear similar (but not statistically significant) trend was found in the coupling strength analyses (Figure 7; Table 2). These findings suggest that substances such as gases or volatile olfactory cues that could pass through the mesh partition, but not in the sealed tube, act additively with substrate borne vibrations to mediate social synchronization. This premise is consistent with earlier studies showing that airflow is sufficient for social synchronization among bees in separated compartments (Moritz and Kryger, 1994; Siehler and Bloch, 2020). Phase drift toward the forages as well as coupling strength appeared somewhat higher for bees in cages separated by a single compared to double mesh partition but neither of these trends crossed the statistical significance threshold (Figures 6, 7). Additional studies with finer analyses are needed for testing the significant of this trend which if supported, is consistent with the hypothesis that short distance interactions such as tactile information or contact pheromones also contribute to social synchronization in honey bees.

Our findings revealed an incredibly strong effect of a small number of foragers on the activity rhythms of young bees, but not vice versa. Using our sensitive ICON pipeline we found no evidence that young bees, which typically show weak activity rhythms, can influence the phase of older sister foragers. These findings lend credence to a model stating that surrogates of forager activity entrain the rhythms of nest bees, and not vice versa in large, freely foraging colonies (Bloch et al., 2013; Siehler et al., 2021). Given that the foragers are entrained by day-night oscillations in light intensity and ambient temperature, entrainment by surrogates of their activity assures that nest bees that spend most, or even all, of their time inside the dark and tightly regulated cavity of the nest, have their internal clocks in phase with the ambient environment. Thus, young bees can precisely coordinate their daily activity with that of foragers and can time their first exit from the nest for orientation flights in which they learn about their nest environment (Capaldi et al., 2000; Degen et al., 2015). The results presented here, together with an earlier study showing that young bees can be socially synchronized to a common phase (Siehler et al., 2021), show that young bees are extremely sensitive to socially synchronizing time cues. A small number of foragers were able to synchronize the daily activity rhythms of all the bees on the same tray, even if their cages were not connected (Figure 4F). Tube connection between adjacent cages significantly improved social synchronization. This is remarkable given that each cage housed only a single bee, and the tube connection enables limited propagation of activity-related cues generated by this single individual.

To sum, the results presented here together with our recent study with callow bees (Siehler et al., 2021), provide important support for the self organization model stating that surrogates of activity mediate social synchronization of honey bees to a common phase (Bloch et al., 2013). Our studies using arrays of separated cages also show that honey bee workers are extremely sensitive to the forager social time cues. Social synchronization is mediated by multiple modalities that include substrate borne vibrations, volatile cues, and perhaps also close contact interactions (Moritz and Kryger, 1994; Siehler and Bloch, 2020; Siehler et al., 2021). Taken together, this evidence suggests that the circadian system of honey bees evolved remarkable sensitivity for entrainment by non-photic, non-thermal, time giving cues enabling them to tightly coordinate their behavior in the constant physical environment of their nests.

## Data Availability Statement

The raw data supporting the conclusions of this article will be made available by the authors, without undue reservation.

## Author Contributions

OS and GB designed the research. OS performed the experiments. SW contributed new analytic tools and analysed data. OS, SW, and GB wrote the manuscript. GB conceived and supervised the research and provided funding. All authors contributed to the article and approved the submitted version.

## Conflict of Interest

The authors declare that the research was conducted in the absence of any commercial or financial relationships that could be construed as a potential conflict of interest.

## Publisher’s Note

All claims expressed in this article are solely those of the authors and do not necessarily represent those of their affiliated organizations, or those of the publisher, the editors and the reviewers. Any product that may be evaluated in this article, or claim that may be made by its manufacturer, is not guaranteed or endorsed by the publisher.
